# Long-term memory predictors of adult language learning at the interface between syntactic form and meaning

**DOI:** 10.1371/journal.pone.0275061

**Published:** 2022-10-03

**Authors:** Diana Pili-Moss

**Affiliations:** Institute of English Studies, Faculty of Education, Leuphana Universität, Lüneburg, Germany; Potsdam University, GERMANY

## Abstract

Recent neurocognitive models of second language learning have posited specific roles for declarative and procedural memory in the processing of novel linguistic stimuli. Pursuing this line of investigation, the present exploratory study examined the role of declarative and procedural memory abilities in the early stages of adult comprehension of sentences in a miniature language with natural language characteristics (BrocantoJ). Thirty-six native Italian young adults were aurally exposed to BrocantoJ in the context of a computer game over three sessions on consecutive days. Following vocabulary training and passive exposure, participants were asked to perform game moves described by aural sentences in the language. Game trials differed with respect to the information the visual context offered. In part of the trials processing of relationships between grammatical properties of the language (word order and morphological case marking) and noun semantics (thematic role) was necessary in order reach an accurate outcome, whereas in others nongrammatical contextual cues were sufficient. Declarative and procedural learning abilities were respectively indexed by visual and verbal declarative memory measures and by a measure of visual implicit sequence learning. Overall, the results indicated a substantial role of declarative learning ability in the early stages of sentence comprehension, thus confirming theoretical predictions and the findings of previous similar studies in miniature artificial language paradigms. However, for trials that specifically probed the learning of relationships between morphosyntax and semantics, a positive interaction between declarative and procedural learning ability also emerged, indicating the cooperative engagement of both types of learning abilities in the processing of relationships between ruled-based grammar and interpretation in the early stages of exposure to a new language in adults.

## Introduction

The role of nondeclarative and declarative learning abilities in adult second language (L2) learning and processing has received increasing attention in recent years. Nondeclarative learning abilities are thought to be available to the infant from the earliest stages of development [1], support skill learning and unconscious learning processes that occur as a result of extended repeated stimuli exposure in the environment, and engender learning that is context-specific and, once consolidated, relatively stable over time. At neuroanatomical level nondeclarative memory functions have been related to activation of cortical-striatal pathways and the cerebellum (particularly procedural learning ability [2]), as well as sensory-cortical areas (particularly statistical learning ability [3]). In contrast to nondeclarative learning abilities, declarative learning abilities undergo rapid development from late childhood, since the neural structures that support them (e.g., hippocampus, medial temporal lobe) mature comparatively later both anatomically and functionally [4, 5]). Declarative learning offers a particularly efficient learning route because it is fast (learning can occur with limited exposure to the stimuli [6, 7]) and flexible (i.e., learning can be transferred to a different context [8]). Although declarative learning does not require awareness in order to occur [9], it typically characterizes conscious learning processes.

### Declarative and procedural learning abilities in adult L2 learning

Declarative learning ability is generally assumed to particularly support adult L2 learning and processing. A robust relationship between late L2 acquisition and reliance on declarative and semantics-based learning strategies has been posited in different, though largely convergent, theoretical frameworks. For example, the Shallow Structure Hypothesis [10] maintains that L2 processing, particularly in comprehension, relies to a larger extent on ‘semantic, pragmatic, probabilistic or surface-level information’ (p. 694) compared to L1 processing, which by contrast would be characterized by deep, structural processing of morphosyntactic information. More recently, Verissimo et al. [11] have showed that which of the two processing routes is relied on in L2 processing is further modulated by age of acquisition, with early (i.e., child) learners displaying processing patterns more closely resembling native processing compared to late (i.e., adult) learners.

From a cognitive second language acquisition (SLA) perspective, proponents of usage-based approaches, as well as skill-acquisition approaches [12, 13] have argued for a main role of declarative learning or declarative knowledge in the early stages of adult exposure to a new language. As the amount of language input increases, reliance on declarative learning/knowledge would progressively decrease and give way to language proceduralization. Specifically, usage-based approaches have argued for proceduralization of L2 form-meaning pairings (constructions) across different domains of linguistic competence (vocabulary, phonology and morphosyntax, etc.,..).

Declarative/Procedural (DP) models [13, 14], see also Paradis [15] for a slightly different conceptualization, have also underscored the role of long-term memory in adult L2 learning. Similar to usage-based approaches, Ullman’s version of the model maintains that declarative and procedural long-term memory are both implicated in adult L2 learning processes, with declarative memory playing a more prominent role at early stages of L2 exposure (or at lower L2 proficiency), and procedural learning having a more substantial role as exposure to the language and language proficiency progress. However, unlike usage-based approaches, the DP model also proposes that the engagement of declarative and procedural memory is modulated by the type of linguistic structure to be learned. In Ullman’s model, declarative memory is posited to particularly support the acquisition of language chunks, irregular forms and vocabulary, whereas procedural memory would support learning of rule-based grammar patterns across morphosyntax (for example word order, case and agreement marking) and phonology. Overall, as a consequence of the combined moderating effect of amount of input and type of linguistic target, Ullman’s DP model predicts engagement of adult declarative memory in the learning of both vocabulary and ruled-based grammar early on during L2 exposure (or at low L2 proficiency levels). For increasing amounts of L2 input (or at higher L2 proficiency levels) declarative learning ability would continue to support development in vocabulary, whereas procedural learning ability would increasingly support learning of rule-based grammar.

In the light of current theoretical frameworks, the present study investigates the role of different long-term learning abilities in the initial stages of adult comprehension of novel linguistic structures where grammatical properties of noun phrases affect sentence interpretation. One such context is represented by thematic linking, the set of rules linking the position of a noun phrase in the sentence or its morphological marking to its thematic interpretation (determining, for example, whether the noun should be interpreted as an agent or a patient). In view of the posited relationship between type of long-term memory and learning of specific aspects of language [14], ‘interface’ constructions of this type offer an interesting testing ground to gauge the extent to which declarative learning ability, largely responsible for the processing of meaningful stimuli, and procedural learning ability, believed to underpin processing of morphosyntactic regularities, engage in the early stages of the exposure to a new linguistic system.

A specific point of interest is the extent to which declarative and procedural learning abilities interact in supporting learning of interface structures. The importance of examining interactions between cognitive abilities has been recently advocated both in cognitive psychology [3, 16] and in the SLA literature [17, 18] and it is motivated by established neurocognitive evidence showing that the declarative and nondeclarative memory systems do not operate exclusively or in isolation but process environmental stimuli by learning ‘in parallel’, as well as interacting cooperatively and competitively during stimuli processing [3, 14, 19, 20]. Thus, also at behavioural level, it seems justified not only to consider the extent to which cognitive abilities relating to long-term memory functions are individually engaged in the learning of novel constructions but also to examine how they interact over the time course of language processing.

In order to provide a background to the present investigation, the next section will review a selection of studies that have examined relationships between long-term memory abilities and L2 outcomes in adult learners. Subsequently, I will turn to the current study that investigates the role of declarative and procedural cognitive abilities in adult L2 sentence comprehension in contexts where the correct thematic interpretation of subject and object noun phrases is determined by their morphosyntactic properties.

### Declarative and procedural learning abilities as predictors of adult learning of novel L2 grammar

In this short review I will distinguish between studies that have investigated the role of long-term memory assessing the acquisition of L2 grammar ‘out of context’ (i.e., where linguistic forms were assessed in isolation and with no relation to a context where they could be interpreted) and studies that have probed the acquisition of L2 form-meaning relationships (i.e., where learning of novel grammar constructions was assessed ‘in context’ relative to their relationship to semantics).

The first set of studies have mostly employed miniature languages designed to reflect natural language morphosyntax and have typically assessed the role of cognitive learning abilities in predicting accuracy in grammaticality judgment tests administered aurally and without the support of a visual context. In these conditions, grammaticality judgment tests mainly probed sensitivity to the grammaticality of morphosyntactic patterns (e.g., word order, case marking), but did not provide information about learning of potential relationships between morphosyntax and interpretation. Some of these studies have employed Brocanto2, a paradigm where participants learn a novel miniature language with natural language characteristics in the context of a computerized gaming environment and where they are asked to either perform moves that correspond to aural descriptions in the language, or orally produce sentences in the language that correspond to moves they see on-screen [21–23].

Overall the results of these studies have converged in finding that declarative learning abilities (assessed via behavioural verbal and visual declarative memory measures) tended to positively predict learning of morphosyntax at early stages of exposure (see also Hamrick [24] for similar results obtained using a different artificial language paradigm). However, after extended exposure (or after periods of no exposure [24]) morphosyntactic learning (particularly word order) became positively related to learners’ procedural learning ability measured by probabilistic learning tasks or implicit sequence learning tasks [21–23, 25].

Turning to studies that assessed sentence interpretation, Pili-Moss et al. [18] investigated Brocanto2 online comprehension data collected but not analysed in Morgan-Short et al. [23]. They found that, in contrast with the grammaticality judgment test analysis in the original study, declarative learning ability was a consistently significant predictor of online sentence comprehension throughout language exposure (across 72 game blocks) and procedural learning ability was not significantly related to accuracy in comprehension at the end of practice (although it strongly predicted comprehension automatization as game practice progressed). Pili-Moss et al. speculated that discrepancies with previous studies and the role of declarative learning ability in consistently supporting accuracy at later stages of exposure could be task-dependent [19], specifically at least partially due to the enhanced visual and semantic processing associated with the online game task compared to the aural grammaticality judgment test. Divergent results between grammaticality judgment test and sentence comprehension measures in the Brocanto paradigm were also evidenced for children at early stages of exposure (after 6 game blocks) [26]. Here grammaticality judgment test scores were positively related to procedural learning ability measured by an alternate serial reaction time task, whereas sentence comprehension was mainly predicted by vocabulary learning ability and a composite of verbal and visual declarative learning ability throughout practice, although to a lesser extent participants with stronger procedural learning abilities also tended to attain higher accuracy towards the end of practice.

Other adult miniature language studies that employed comprehension-based task as outcome measures (e.g., picture-based wug tests or forced-choice comprehension tasks) considered learning of word affixation rules and found that both declarative and procedural learning ability predicted accuracy but that relationships were mediated by morphological rule complexity [17, 27] or that statistical learning ability predicted language outcomes [28]. Specifically, Antoniou, Ettlinger, & Wong [17] found that after limited exposure to novel inflexional patterns (192 trials in one session) declarative learning ability predicted learning of complex rules (affixation with vowel harmony and vowel reduction), whereas procedural learning ability predicted learning of simple rules (simple affixation). Given that sentences in the Brocanto studies instantiated complex rule patterns (they included multiple rules) the results in Antoniou et al. align with those of Brocanto studies in underscoring an early main role of declarative learning ability in the learning of complex novel morphosyntax.

Notwithstanding methodological differences, it should be noted that discrepancies between grammaticality judgment test and comprehension task results have also emerged for natural languages in studies analysing the role of cognitive aptitudes in learners’ L2 ultimate attainment (the highest L2 proficiency level reached by learners after extended periods of residence in a country where the L2 is the primary language used for communication). Generally, studies have found positive relationships between explicit language aptitude (the ability to learn language rules and patterns explicitly) and the L2 grammar attainment of late acquirers measured via a grammaticality judgment task [29, 30]. However, some studies did not [31].

Overall, particularly concerning the body of training studies developed in the Brocanto game paradigm, it seems that, independently of the age of the learners, syntactic outcomes assessed via sentence comprehension tasks during the game practice related significantly to behavioural measures of declarative memory. When grammaticality judgment test outcomes are considered, language outcomes were modulated by additional effects of amount of input (or level of proficiency) and age. A substantial role for declarative learning ability at early stages of exposure is also confirmed for complex morphological rules assessed in tasks probing the establishment of novel form-meaning relationships. However, success in tasks involving language interpretation is not exclusively supported by declarative learning abilities. Training studies investigating adult learning of simple affixation rules [17, 27, 28], as well as child studies investigating learning of morphosyntax [26], have also indicated a role of procedural or statistical learning ability in supporting language development after relatively short exposure phases.

### The current study

The current study investigates the extent to which declarative and procedural learning abilities support adult learning of thematic linking in the Brocanto paradigm and interact during language processing by examining aural sentence comprehension during the game practice. In order to minimize confounds due to the strong tendency of adult L2 learners to rely on declarative learning strategies as a faster and more efficient route to process linguistic input for comprehension, it was deemed important to identify learning conditions where contextual cues could not be used as shortcuts to L2 comprehension.

To this end, and unlike previous Brocanto studies [18], two separate types of online game trials were identified and compared; moves presenting reversible actions, for which learning of relationships between NP morphosyntax and thematic content was necessary for correct sentence interpretation, and trials where accurate interpretation could be arrived at by exploiting visual and contextual cues, so that learning of relationships between morphosyntax and the thematic interpretation of NPs was not key to accuracy. The research questions were formulated as follows:

RQ: To what extent do declarative and procedural learning abilities support sentence comprehension in the early stages of L2 practice (1) when processing of form-meaning relationships is essential for interpretation, as opposed to (2) when interpretation can rely on disambiguating contextual cues?

Based on previous studies employing the same experimental paradigm [18] and since language outcomes were assessed via an online comprehension measure in the early stages of exposure, it was hypothesized that in both case (1) and (2) declarative learning ability would be positively related to accurate sentence comprehension. Additionally, since procedural learning ability has been posited to have a specific role in the learning of novel grammar patterns [14], a further hypothesis was that it would also support the acquisition of pattern-based relationships between morphosyntax and interpretation. In this case, one would expect this effect to emerge more clearly in (1), where processing of relationships between grammar and meaning is mandatory, rather than in (2), where the context provides sufficient cues to support comprehension. The study’s findings confirmed both sets of hypotheses.

## Materials and methods

### Participants

The participants were 36 L1 Italian young adults (17 females; *M* = 22, *SD* = 3.7; range 18–31) with no history of learning differences or hearing impairment and normal or corrected to normal vision. Except for one male and one female (who had completed secondary education and were in work), all participants were university students at the time of testing. All had had formal instruction in English as a second language for periods >10 years, reported to have had instruction on average in 2.5 languages (range 1–5) and received monetary compensation for their participation. The study was approved by the Ethics Committee of Lancaster University and informed written consent was obtained by all participants.

### Vocabulary training

The study design included three sessions on separate subsequent days lasting about 40–45 minutes, 50 minutes and 60 minutes respectively, during which the participants were exposed to BrocantoJ in the gaming environment. In the Vocabulary Training phase participants learned the 12 BrocantoJ lexical items (Table 1). All vocabulary items were introduced aurally by the researcher without using translations and in association with a corresponding static picture (game tokens, adjectives and directions) or animation (moves). For a detailed description of the vocabulary training procedure see Pili-Moss [26]. Vocabulary training was followed by a test where participants had to reach a criterion of 100% correctly identified word/visual associations in order to proceed to the subsequent stage of the experiment. If criterion was not reached, further instruction on the set/s where mistakes were made was provided, followed by a repetition of the vocabulary test (all items). The procedure was repeated for a maximum of four times. Vocabulary testing was repeated at the beginning of each subsequent session to ensure vocabulary knowledge had been retained. A score of vocabulary learning ability (VocLearn) was obtained for each participant standardizing (*z*-scores) the sum of errors in the vocabulary test across the three sessions multiplied by -1.

**Table 1:** Vocabulary items in BrocantoJ.

BrocantoJ	Category	Meaning
blomi	Noun	the ’blomi’ token
nipo	Noun	the ’nipo’ token
pleca	Noun	the ’pleca’ token
vode	Noun	the ’vode’ token
trose	Adjective	round
neimo	Adjective	square
klino	Verb	move (intrans.)
nima	Verb	capture (trans.)
yabe	Verb	release (trans.)
prazi	Verb	switch (trans.)
noika	Adverb	vertically
zeima	Adverb	horizontally
ri	Preposition	nominative case
ru	Preposition	accusative case

### Passive exposure and game practice

After vocabulary training the participants listened to full sentences in BrocantoJ, in association to a corresponding visual animation exemplifying the corresponding move in the game, but did not actively play the game (Passive Exposure). The passive exposure set consisted of 144 BrocantoJ sentences administered in 6 blocks. Block 1 was administered in Session 1, Block 2 and 3 were administered in Session 2 and Blocks 4, 5 and 6 were administered in Session 3. After each passive exposure video (about 4 minutes), the participant had the opportunity of playing a game block (Game Practice; 120 BrocantoJ stimuli in 6 blocks, 20 items per block). In both passive exposure and game practice the order of presentation of the sentence stimuli was the same for all participants. In the game practice participants were given the instruction to listen well to the words in the new language, and then make the move they thought the words were describing as fast as they could. After making their move, they were immediately given feedback on screen in the form of the words ’correct’ or ’incorrect’. The next stimulus was presented immediately afterwards or after 60 seconds in case of no response. Unbeknownst to the participants the computer program created a by-trial online record of their moves and running score that provided an online overall measure of accuracy of language comprehension at item level. Participants were shown a percentage accuracy score at the end of each game block.

### The language BrocantoJ

BrocantoJ is a version of BROCANTO2 [32], has 12 lexical items and 2 prepositional case markers and displays the morphosyntax of Japanese declarative main sentences (Table 1 and example 1).

(1) (Adj-Noun-NOM marker) - (Adj-Noun-ACC marker)–Adv–Verb

**Fig 1:**
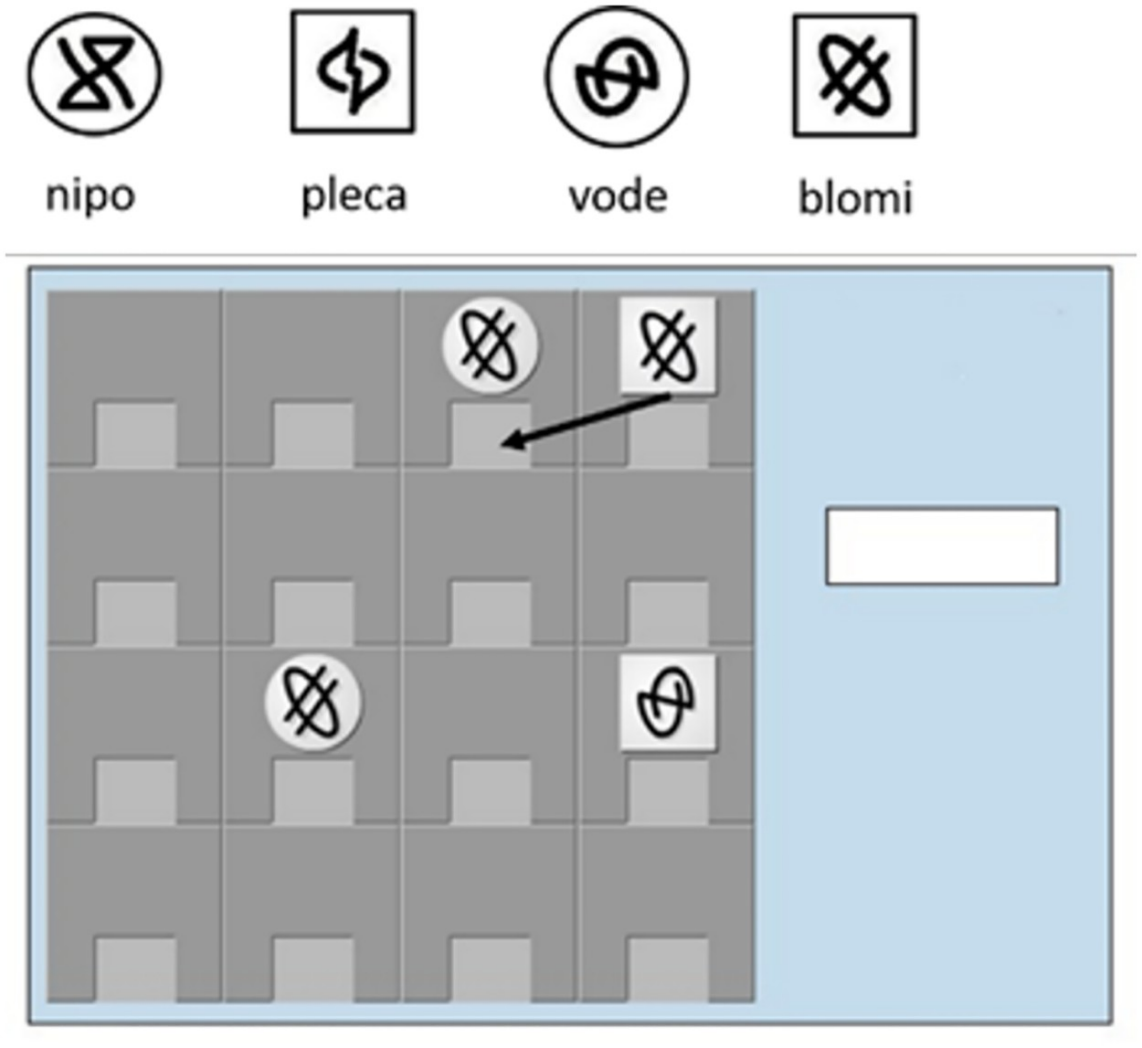
Game tokens and constellation corresponding to sentence (2). Adapted from Pili-Moss [33: p.117].

Example (2) and Fig 1 illustrate a BrocantoJ sentence and the corresponding game constellation

(2) Trose  blomi ri     neimo blomi ru     zeima      nima
    Round blomi NOM  square blomi ACC  horizontally  capture
  ‘The round blomi piece captures the square blomi piece horizontally’

**Fig 2:**
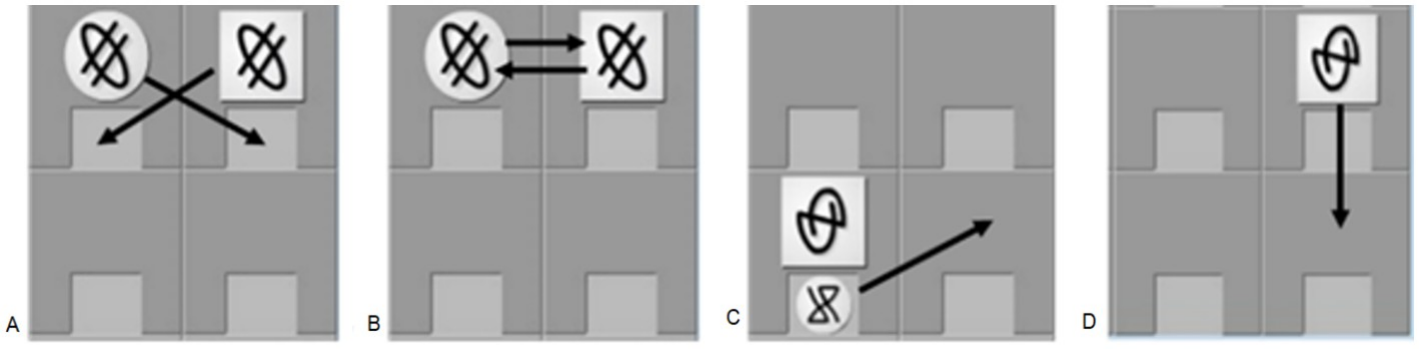
Symmetric Moves Corresponding to the Verbs Nima (a) and Prazi (b), and Asymmetric Move Corresponding to the Verbs Yabe (c) and Klino (d). Adapted from Pili-Moss [33: p.117].

In total the game has four moves corresponding to the language’s four verbs: (a) ’move’ (*klino*, a simple one-square move; Fig 2d); (b) ’capture’ (*nima*, a token is captured by a net generating from the internal square of an adjacent token’s position and is dragged there, Figs 1 or 2a); (c) ’swap’ (*prazi*, two adjacent tokens swap positions with one of the two moving first/ initiating the move, Fig 2b); and (d) ’release’ (*yabe*, a captured token is released in an adjacent free position, Fig 2c).

As Fig 2a and 2b illustrate, two of the three moves expressed through transitive verbs (’capture’ and ’swap’) visually correspond to *symmetric* configurations. This means that given an initial game constellation each of the two tokens could capture/be captured or actively swap/be swapped). In this context the visual information offered by the initial constellation does not provide cues as to which of the two tokens will move (or move first), and accurate processing of the mapping between thematic functions and syntactic positions or between thematic functions and case markers is key.

By contrast, 2c and 2d (’release’ and ‘move’) exemplify *asymmetric* configurations. In both scenarios the visual information provided in the initial constellation unambiguously indicates which token will move, i.e., provided that the type of move, the tokens and the direction of movement have been correctly identified, an understanding of the morphosyntactic form-meaning relationships in the sentence is not strictly necessary. In this cases, accurate performance could entirely rely on the application of general heuristic strategies.

### Cognitive measures

#### Declarative learning ability

**Fig 3:**
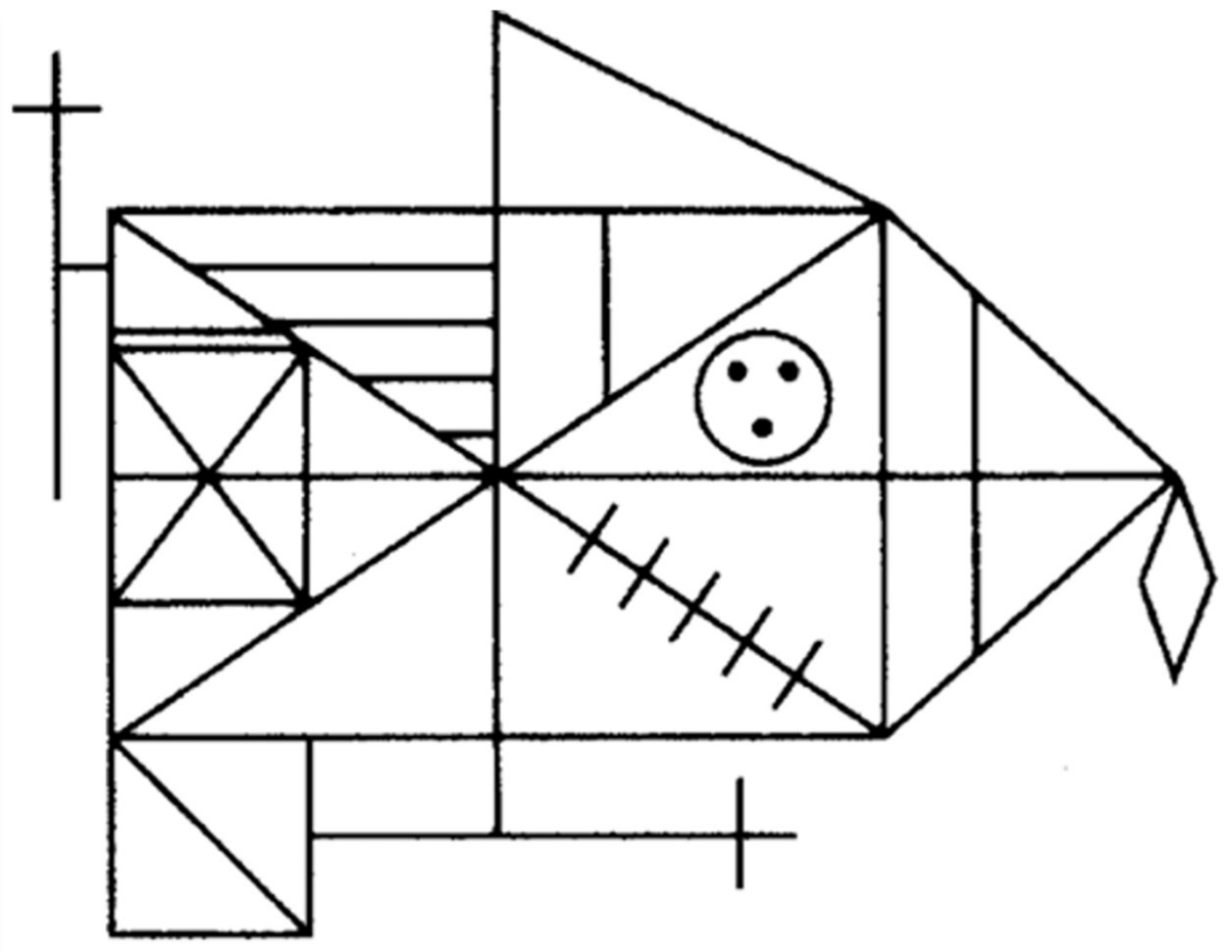
Rey-Osterrieth complex figure.

Visual Declarative Learning Ability was indexed by performance on the Rey-Osterrieth Complex Figure Task [34, 35] (Fig 3); interrater reliability coefficients between .93 and .96, and intrarater reliability coefficients between .93 and .98 [36]. In the task the figure was presented to the participants who were given about 5 minutes to draw a copy on paper.

After a 15 minute delay, during which the participants were occupied with the first part of a verbal declarative memory task and two working memory tasks, they were asked, with no previous warning, to reproduce the figure again from memory in as much detail as possible and given up to 10 minutes to complete the task. The recall drawing was scored based on the reproduction of 18 elements on a two-point scale (range: 0–36). Two points were given if the item was placed and drawn correctly, one point if it was either well drawn but placed incorrectly or incomplete but placed correctly, half a point if the item was present but incomplete and placed incorrectly, and zero points if the element was absent or not recognizable [37].

Verbal Declarative Learning Ability was indexed by performance on a story recall [38], designed as an Italian version of the Logical Memory subtest in the Wechsler memory scale; test-retest reliability, *r* = .74 [39]. In this task a short story (57 words) containing 28 information units is read to the participants, who are asked to repeat it immediately afterwards (immediate recall) and after a 15-minute delay (delayed recall). Following Mondini et al. [38], one point was assigned for each accurate information unit in the immediate recall of the story. The procedure was repeated for the delayed recall and the scores of both recalls were averaged. A declarative learning ability composite score was obtained by standardizing and then averaging the scores of the visual and verbal tasks.

#### Procedural learning ability

Procedural learning ability was indexed by performance on an alternate serial reaction time task adapted from a previously employed serial reaction time paradigm [40]. Previous studies investigating the reliability of this task have found a test-retest *r* = .45 [41], Cronbach α values between .75 and .79 (RT scores) and between .69 and .75 (accuracy scores) [42], as well as a Spearman-Brown split-half reliability index of .42 [25]. Participants saw four squares arranged in a diamond shape configuration on screen and were asked to immediately react to the changes in the position of a smiley by pressing the corresponding buttons on a game controller. Unbeknownst to the participants, the visual stimuli sequence was not random but followed the pattern 1 r 2 r 4 r 3 r, where sequence positions alternated with random positions [43].

After a familiarization phase, there were 8 experimental blocks (80 trials each), with the possibility for the participant of taking brief self-managed breaks between blocks. The blocks and the trials in each block were administered to all participants in the same order, and each block was preceded by 5 warm-up trials. The last screen in each block gave the participants feedback about their accuracy (percentage correct) and about the speed of their performance in the block. The participants were told to play the game trying to press the correct controller button as fast as they could. They were also told to check the scores at the end of the block and try to keep an accuracy score around 92% [44], whilst at the same time trying to improve their speed score as the game progressed.

Procedural learning ability was operationalized as learning of the fixed pattern in the alternating stimuli sequence, as evidenced by increasingly reduced reaction times on sequence trials compared to random trials and greater inaccuracy on random trials compared to sequence trials [43, 45]. The data were first reduced in Excel excluding practice trials, warm-up trials, incorrect trials, and trials that were the final element in ’trills’ (e.g., 212) or in ’repetitions’ (e.g., 222) [43, 44].

For the RT-based measure, the median RT values in milliseconds were calculated separately for sequence trials and random trials for each participant’s block. In both data subsets the scores from Block 1 to Block 4 and the scores from Block5 to Block 8 were averaged obtaining an A and a B score respectively. The difference between A and B (RT Gain) reflected the change in reaction times from the first half to the second half of the training. To obtain a final measure of procedural learning ability based on RTs, RT Gains from random trials were subtracted from RT Gains from sequence trials, with higher positive differences indicating better sequence learning.

The (in)accuracy measure dataset also included incorrect items. The number of inaccurate responses (errors) was calculated for each participant per type of trial and block and then averaged across blocks. The difference between the average number of errors in random trials and in sequence trials provided a measure of sequence learning, with larger positive differences indicating better sequence learning. Finally, a composite measure of procedural learning ability (Proc) was obtained standardizing and then averaging the two components.

### Other cognitive covariates

#### Phonological loop

Phonological short-term memory was indexed by performance on a Forward Digit Span; test-retest reliability *r* = .81 [46]. The researcher read to the participant a series of digit sequences of increasing length (from 3 to 9 items) with monotone intonation and at the rate of about one second per digit. The participants were instructed to subsequently to repeat the full sequence, and, if they could recall it correctly, were read a sequence one digit longer. In case of errors, a second sequence of the same length was presented. The test ended when errors on two consecutive lists of the same length occurred.

#### Working memory

Working memory was indexed by performance on a Backward Digit Span, a task similar to the previous one but where participants are asked to reproduce sequences backwards (test-retest reliability *r* = .82 [46]). Unlike the Forward Digit Span, the Backward Digit Span is considered to be a measure of the Central Executive because it not only requires the participant to retain information in short term memory for immediate repetition, but also to perform an operation (order reversal) before reproduction [47]. For both Forward and Backward Digit Span final scores corresponded to the number of elements in the longest correctly recalled sequence. Materials as well as administration and scoring protocols were taken from a previous study that also provided recent normative data for the Italian adult population [48].

#### Verbalized explicit knowledge

In order to gauge explicit knowledge of BrocantoJ at the end of the experiment, participants were interviewed using a 4-item questionnaire and asked to report what they had noticed about the new language (S1; intra-rater Cohen’s *k* = .65). Adapting Rosa and Leow’s methodology [49] a language awareness score was obtained as follows: (1 point) reports to have noticed nothing in particular; (2 points) reports noticing the presence/absence of specific words; (3 points) reports noticing the presence/absence of a single specific word and refers to its position in the sentence; (4 points) reports that there is an order involving domains larger than a single word but does not provide examples; (5 points) reports that there is an order in domains larger than a single word and provides examples; (6 points) provides a complete example of the sentence word order in the new language.

## Results

### Data analysis

The inferential statistics employed binomial linear mixed-effect models (*glmer* function with maximum likelihood, Laplace approximation, from the *lme4* package in R [50, 51]. The variables considered included declarative (Decl) and procedural (Proc) learning ability, Session and Block. Other covariates entered in the model were measures of working memory, phonological short-term memory, attainment in vocabulary testing (VocLearn), verbalized explicit knowledge (VerbExpl), age at testing, gender, years of schooling, whether participants attended university, number of additional languages the participants had been instructed in or had had extended exposure to (> 6 months) and the number of days between session 1 and session 2 (S1S2) and session 2 and session 3 (S2S3). Interactions between Decl and Proc and between these and Session or Block were also investigated. All continuous variables were centered and the ID variables were also standardized.

Fixed effects (including interactions) were added one at a time successively comparing nested models using the likelihood ratio test and the Akaike Information Criterion (AIC). Fixed effects were included in the model if the model converged and the effect statistically significantly improved the model’s fit. Random effects were explored in the same way starting from random effects on intercepts (items and participants) and followed by random effects on the slopes of the fixed effects. The interpretation of the magnitude of effect sizes (OR) follows Cohen [52] and Sánchez-Meca, et al. [53]. Inspection of the models’ Q-Q plots indicated that the distribution of residuals was approximately normal. Given the relatively low condition number, multicollinearity between the predictors was not deemed to pose an issue in either the symmetric or the asymmetric datasets. The complete dataset and R code are available at https://osf.io/nh4cx/.

### Descriptive and inferential statistics

Table 2 reports descriptive statistics relative to the accuracy outcomes of symmetric trials (Cronbach *α* = .94) and asymmetric trials (Cronbach *α* = .99), whereas Tables 3 and 4 respectively show raw scores of the components of declarative and procedural learning ability, as well as Pearson’s correlations between the main cognitive predictors and accuracy in symmetric and asymmetric contexts.

**Table 2:** Mean accuracy of symmetric and asymmetric game trials.

	*M* (*SD*)	*S*.*E*.	%	*Total*
Symmetric	29.7 (14.0)	2.3	42.3%	72
Asymmetric	30.8 (10.2)	1.7	65.5%	48

**Table 3:** Raw score means relative to the components of the cognitive main predictors.

	*M* (*SD*)	*SE*
Decl (visual)	109.71 (11.9)	1.94
Decl (verbal)	114.84 (11.3)	1.83
Proc (Acc)	2.40 (1.5)	0.25
Proc (RT)	-5.63 (23.5)	3.92

**Table 4:** Correlations between main predictors and measures of accuracy.

	Decl	Proc	Symmetric	Asymmetric
Decl	-	-.008	.378	.442
Proc		-	.141	.014
Symmetric			-	.901

RQ1 asked the extent to which declarative and procedural learning ability predict accurate comprehension in trials where establishing a relationship between the word order (or morphology) of the language and the thematic functions of its noun phrases was necessary for accurate sentence interpretation (symmetric contexts). The model in (1) provided the best fit accounting for about 46% of the variance in the symmetric data subset (Table 5):

(1) ACC ~ (1|PART) + (1|ITEMS) + DECL * PROC * SESSION + VOCLEARN + VERBEXPL

**Table 5:** Generalized mixed-effects model of the effects of declarative learning ability, procedural learning ability, session and ID covariates on accurate performance in symmetric contexts (R^2^Δ = .46; marginal R^2^Δ = .24, C.N. = 1.61).

				Wald CI (95%)		
Fixed effects	*β*	*SE*	*z*	lower	upper	*p*
(Intercept)	-0.47	0.19	-2.47	-0.85	-0.10	.014*
Decl	0.51	0.24	2.15	0.04	0.98	.032*
Proc	0.14	0.22	0.63	-0.30	0.58	.530
Session	1.24	0.19	6.46	0.86	1.61	< .001***
VocLearn	0.63	0.16	4.01	0.32	0.94	< .001***
VerbExpl	0.45	0.14	3.27	0.18	0.72	.001**
Decl*Proc	0.36	0.34	1.06	-0.30	1.02	.288
Decl*Session	0.37	0.13	2.86	0.11	0.62	.004**
Proc*Session	-0.20	0.12	-1.67	-0.44	0.03	.096
Decl*Proc*Session	0.69	0.19	3.71	0.33	1.06	< .001***

Note.

*p < .05;

**p < .01;

***p < .001.

*N* of valid observations = 2496

**Fig 4:**
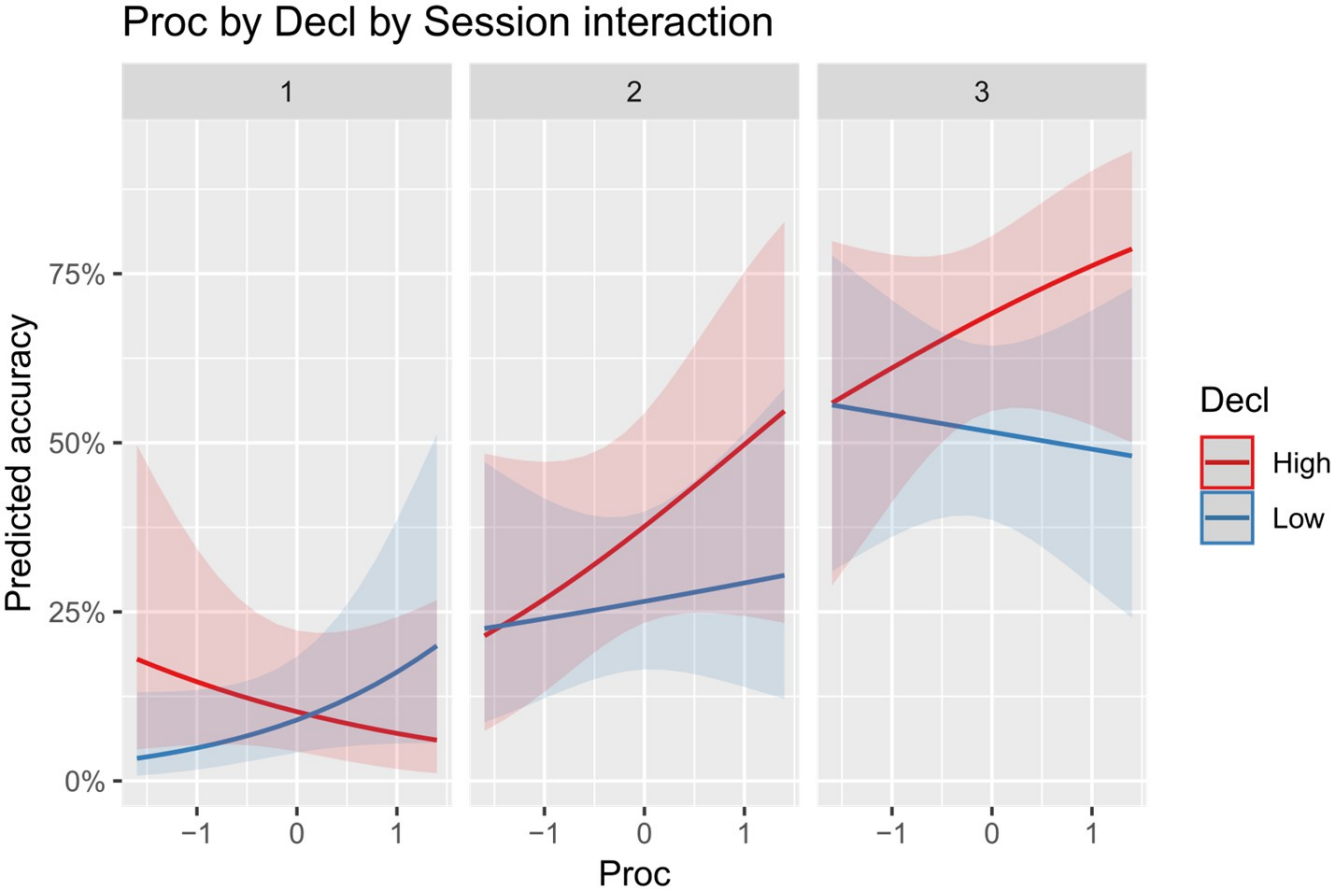
Predicted effect of the interaction between procedural and declarative learning ability on accurate performance in symmetric trials in session 1, 2 and 3.

The model returned a significant positive effect of a Decl by Proc by Session interaction (OR = 1.99, a small to medium effect; Fig 4), and significant conditional effects of a Decl by Session interaction (OR = 1.45, a small effect), of Decl (OR = 1.66, a small effect), VocLearn (OR = 1.88, a small effect) and VerbExpl (OR = 1.57, a small effect), whilst the conditional effect of procedural learning ability was nonsignificant. A plot of the three-way interaction (Fig 4) reveals that, at very initial stages of practice (session 1), higher procedural memory was only beneficial to learners with lower levels of declarative memory and adversely affected learning in higher declarative memory learners. However, this pattern reversed as practice with the language progressed (session 2 and 3) and higher procedural memory increasingly supported learning specifically in strong declarative memory learners.

RQ2 asked the extent to which declarative and procedural learning ability predicted accurate comprehension in trials where visuo-contextual cues were sufficient to disambiguate the thematic function of nominal phrases and, although possible, processing of word order/morphological cues was not necessary for comprehension (asymmetric contexts). The model in (2) and Table 6 provided the best fit accounting for about 35% of the variance in the asymmetric data subset:

(2) ACC ~ (1|PART) + (1|ITEMS) + DECL + PROC + BLOCK + VOCLEARN + VERBEXPL + S1S2

**Table 6:** Generalized mixed-effects model of the effects of declarative learning ability, procedural learning ability, block and ID covariates on accurate performance in asymmetric contexts (R^2^Δ = .35; marginal R^2^Δ = .20, C.N. = 2.07).

				Wald CI (95%)		
Fixed effects	*β*	*SE*	*z*	lower	upper	*p*
(Intercept)	1.01	0.17	5.92	0.68	1.35	< .001***
Decl	0.91	0.20	4.48	0.51	1.30	< .001***
Proc	0.24	0.19	1.26	-0.13	0.62	.207
Block	0.42	0.08	5.08	0.26	0.58	< .001***
VocLearn	0.41	0.12	3.32	0.17	0.65	< .001***
VerbExpl	0.43	0.11	3.85	0.21	0.65	< .001***
S1S2	0.46	0.13	3.66	0.21	0.71	< .001***

Note.

***p < .001.

*N* of observations = 1664

Unlike symmetric contexts, the model shows that the likelihood that single asymmetric trials were accurate was positive and significant. This indicates that, as is to be expected, comprehension in asymmetric trials was on average easier, compared to symmetric trials. The model also returned significant positive effects of Decl (OR = 2.48, a medium effect), VocLearn (OR = 1.51, a small effect), VerbExpl (OR = 1.54, a small effect), Block (OR = 1.52, a small effect), as well as S1S2 (OR = 1.58, a small effect), showing that a larger number of days between the first two sessions was associated to better learning. Again, the effect of procedural learning ability was positive but not significant.

## Discussion

The research questions asked to what extent domain-general declarative and procedural cognitive abilities supported development in the sentence comprehension of a novel miniature language. The present exploratory study investigated two cases: (1) the case in which accurate comprehension could only depend on learning of correspondences between morphosyntax and interpretation (RQ1), and (2) the case in which comprehension could rely on contextual (extralinguistic) cues, so that costly morphosyntactic processing was not key to disambiguation and could be by-passed (RQ2). The results revealed that in both cases declarative learning ability was significantly positively associated to sentence comprehension and that accuracy significantly improved across training. These results confirm the findings of previous training studies that have investigated the development of second language comprehension in adults [18] and in general align with the findings of previous miniature language studies [23, 24] and with the theoretical prediction of a strong role of declarative learning strategies in adult processing of a novel language, particularly in its early stages [12–15].

Another variable that was significantly related to better sentence comprehension in both conditions was the participants’ ability to efficiently learn the BrocantoJ vocabulary items as indexed by the overall attainment across the three vocabulary tests. Better learning of BrocantoJ vocabulary arguably supported online retrieval of word-meaning associations and thus more efficient processing of sentence meaning. The ability to consciously learn, retain and retrieve arbitrary audio-visual (cross-modal) associations would also depend on declarative memory functions. However, it should be noted that this measure was obtained by adding scores across the three testing sessions, hence additional variables, such as the effect of sleep between sessions, may have confounded it.

A further element that emerged in both conditions, and that is related to explicit- declarative learning ability, is that learners that demonstrated a greater capacity to verbalize linguistic rule patterns they had noticed during language passive exposure or practice (independently on whether these were fully correct) were also significantly more likely to correctly interpret BrocantoJ sentences. Verbalized linguistic knowledge has been related to awareness of linguistic regularities [54], although the opposite is not necessarily true, as some participants may acquire explicit language knowledge they are not able to verbalize [55], possibly due to learners’ differences in meta-linguistic abilities. Indeed, increasingly structured reporting of linguistic regularities indicates not only awareness that linguistic regularities existed but also suggests access to progressively more sophisticated levels of meta-linguistic representation obtained via analysis and generalization of explicit linguistic knowledge. Overall, the findings indicate that, across conditions, standardized behavioural measures of declarative learning ability, as well as other indexes of explicit learning and meta-linguistic ability (ability to learn BrocantoJ words, ability to verbalize BrocantoJ linguistic regularities) were significantly positively related to adults’ ability to comprehend aural sentences in a novel miniature language in real time after a relatively short exposure phase.

A variable that appeared to be positively associated to comprehension in the asymmetric condition (RQ2), but not in the symmetric condition (RQ1), was the number of days between session 1 and session 2. The finding that a larger number of days between the first two sessions was associated to better outcomes could be generally related to a positive effect of sleep in supporting declarative long-term memory consolidation in adults [56]. However, why the effect of consolidation emerged only in the asymmetric condition remains unclear.

As a main effect, procedural learning ability did not show strong positive associations with accuracy in either the symmetric or the asymmetric condition. This is in line with the results of previous studies that did not find significant relationships between procedural learning ability and language outcomes in the early stages of adult exposure to novel linguistic stimuli [18], as well as with the expectation that a more extended exposure to language stimuli would be needed for the effects of procedural learning ability to emerge in adults at behavioural level, compared to declarative learning ability [14]. By contrast, only in the symmetric condition, a three-way Decl by Proc by Session interaction was a significant positive predictor of accurate comprehension. Visualization of the interaction showed that, specifically in strong declarative learners, higher procedural learning ability was associated to significantly better comprehension outcomes, particularly towards the end of language practice. Overall, the best sentence comprehension outcomes were achieved by individuals with high levels of both declarative and procedural learning ability in the final practice session.

Considering that accurate sentence comprehension in the symmetric condition strictly depended on the correct establishment of form—meaning relationships between noun phrase positions (or morphology) and noun phrase thematic interpretation, it can be concluded that high levels of both types of long-term memory abilities were needed for effective processing and learning of these relationships. By contrast, in the asymmetric condition, where, although possible, processing of morphosyntactic stimuli for the purposes of interpretation was arguably discarded in favour of less costly reliance on contextual cues, positive interactions between declarative and procedural learning ability did not emerge.

The positive interaction between declarative and procedural learning ability in the early stages of L2 learning apparently mirrors similar findings in at least one previous Brocanto study [18] where, unlike in the present study, items were analysed without categorizing them in symmetric and asymmetric. However, it is important to note that, in that case, the positive interaction was found modelling data relative to the coefficient of variation (CV), a reaction-time based measure of language automatization [57] and that no interactions between declarative and procedural learning abilities emerged when accuracy in language comprehension was considered.

Although the present study was not designed to discriminate between different theoretical models, the finding of distinct patterns of accuracy predictors in the symmetric and asymmetric conditions is generally compatible with the theoretical predictions made by recent (neuro)cognitive models of second language acquisition including the Shallow Structure Hypothesis [10] and the DP model [14]. As predicted by the Shallow Structure Hypothesis, explicit-declarative strategies that take advantage of semantic and contextual information to support comprehension would be the default processing route for adult second language learners. However, deeper processing of morphosyntactic information would not be precluded in adults and, among other possible factors, may be resorted to depending on specific task conditions [10].

Similarly, DP model accounts would expect the role of procedural learning ability in supporting accurate comprehension to emerge in contexts where learning of rule-based grammar patterns (here properties of word order or case marking) is key to comprehension, rather than in cases in which accuracy does not necessarily index learning of properties of the language morphosyntax. Further, from a DP model perspective, the interaction pattern between declarative and procedural learning ability in supporting accurate sentence comprehension in interface constructions could also reflect a cooperative engagement of declarative and procedural memory in the early stages of the learning of relationships between semantics and structural properties of the language.

An alternative way of accounting for the contribution of procedural learning ability to accuracy could be to interpret it as an index of the learner’s ability to proceduralize (and then automatize) declarative linguistic representations [13, 18, 58]. In this scenario, this would lead to more accurate outcomes because automatized comprehension would progressively free up cognitive resources leading to more efficient online processing of the visual and aural stimuli in the learning environment. It should be noted that the process would include not only morphosyntax but also other aspects of the novel language, such as the correspondences between novel words and their meanings [12]. In this case, rather than reflecting the concurrent contributions of declarative and procedural learning ability in the learning of different aspects of a novel grammar construction (semantic vs. morpho-syntactic), the positive interaction would more generally reflect the early stages of proceduralization of declarative language knowledge across the board. However, if the effects of procedural learning ability on accuracy in strong declarative learners were due to across the board proceduralization, one would have perhaps expected to find them also in the asymmetric dataset.

The present exploratory study has a number of limitations that should be addressed in future research on the role of declarative and procedural learning abilities in the early stages of second/additional language learning. The first aspect to observe is that the training period in the present study was relatively short. Further studies could adopt designs with more extended practice periods in order to provide a more complete picture of the time course of long-term memory ability engagement in early sentence comprehension (for example four sessions over the course of two weeks would allow for closer comparisons with previous similar studies [18, 23, 58]. Ideally, it would also be possible to recruit a larger number of participants. However, it should be noted that a sufficiently large number of test items in the present study ensured that the power to obtain the observed effect size for the main cognitive predictors of interest (declarative learning ability in the asymmetric dataset and the three-way Decl x Proc x Session in the symmetric dataset) was well above the 80% threshold ([92.9, 100.0] and [83.4, 98.7] respectively) [59].

Future research, including research employing neuroimaging methods [60], could also examine more closely the extent to which the early effects of procedural learning ability of the type found here are to be specifically related to processing of rule-based aspects of language rather than to initial language proceduralization phenomena that may apply to newly acquired declarative language knowledge more generally (e.g., across morphosyntax and the lexicon).

From a linguistic perspective, the present study was not designed to tease out the extent to which processing of syntactic (word order) versus morphological cues (case marking) was key to establish and support learning of form-meaning relationship. Further research could employ languages where different types of cues are investigated separately and explore how learning effects are moderated by cognitive individual differences in each case.

## Supporting information

S1 File Debriefing interview. (DOCX)

## References

[1] Nelson CA, Webb SJ. A cognitive neuroscience perspective on early memory development. In: de Haan M, Johnson MH, editors. The cognitive neuroscience of development. London: Psychology Press; 2002. p. 99–125.

[2] Lee JC, Tomblin JB. Procedural learning and individual differences in language. LL & D. 2015; 11(3):215–236. doi: 10.1080/15475441.2014.904168 26190949 PMC4504686

[3] Batterink LJ, Paller KA, Reber PJ. Understanding the neural bases of implicit and statistical learning. Top Cogn Sci. 2019;11:482–503. doi: 10.1111/tops.12420 30942536 PMC7723461

[4] Bauer PJ. Toward a neuro-developmental account of the development of declarative memory. Dev Psychobiol. 2007;50:19–31. doi: 10.1002/dev.20265 18085555

[5] Giedd JN, Blumenthal J, Jeffries NO, Castellanos FX, Liu H, Zijdenbos A, et al. Brain development during childhood and adolescence: a longitudinal MRI study. Nat Neurosci. 1999;2(10):861–862. doi: 10.1038/13158 10491603

[6] Eichenbaum H. The cognitive neuroscience of memory: an introduction. 2nd ed. Oxford: Oxford University Press; 2012.

[7] Squire LR, Dede AJO. Conscious and unconscious memory systems. CSH Perspect Biol. 2015;7. a021667. doi: 10.1101/cshperspect.a021667 25731765 PMC4355270

[8] Reber PJ, Knowlton BJ, Squire LR. Dissociable properties of memory systems: differences in the flexibility of declarative and nondeclarative knowledge. Behav Neurosci. 1996 Oct;110(5):861–71. doi: 10.1037//0735-7044.110.5.861 8918990

[9] Henke K. A model for memory systems based on processing modes rather than consciousness. Nat Rev Neurosci. 2010;11(7):523–532. doi: 10.1038/nrn2850 20531422

[10] Clahsen H, Felser C. Some notes on the shallow structure hypothesis. Stud Second Lang Acq. 2018;40(3):693–706. doi: 10.1017/S0272263117000250

[11] Veríssimo J. Heyer V, Jacob G, Clahsen H. Selective effects of age of acquisition on morphological priming: evidence for a sensitive period. Lang Acq. 2017;25(3):315–326. doi: 10.1080/10489223.2017.1346104

[12] Ellis N. Implicit and explicit language learning: their dynamic interface and complexity. In: Rebuschat P, editor. Implicit and explicit learning of languages. Amsterdam: John Benjamins; 2015. p. 3–23.

[13] DeKeyser RM. Skill acquisition theory. In: VanPatten B, Williams J, editors. Theories in second language acquisition: an introduction. Mahwah, NJ: Lawrence Erlbaum Associates; 2015. p. 94–112.

[14] Ullman MT. The declarative/procedural model. In: VanPatten B, Keating GD, Wulff S, editors. Theories in second language acquisition. (3rd ed., pp. 128–161). Abingdon: Routledge; 2020. p. 128–161. doi: 10.4324/9780429503986-7

[15] Paradis M. Declarative and procedural determinants of second languages. Philadelphia: John Benjamins Publishing Company; 2009.

[16] Kidd E, Donnelly S, Christiansen MH. Individual differences in language acquisition and processing. Trends Cogn Sci. 2018;22(2):154–169. doi: 10.1016/j.tics.2017.11.006 29277256

[17] Antoniou M, Ettlinger M, Wong PCM. Complexity, training, paradigm design, and the contribution of memory subsystems to grammar learning. PLOS ONE. 2016;11(7): e0158812. doi: 10.1371/journal.pone.0158812 27391085 PMC4938220

[18] Pili-Moss D, Brill-Schuetz K, Faretta-Stutenberg M, Morgan-Short K. Contributions of declarative and procedural memory to accuracy and automatization during second language practice. Biling-Lang Cogn. 2020;23(3):639–651. doi: 10.1017/S1366728919000543

[19] Foerde K, Knowlton BJ, Poldrack RA. Modulation of competing memory systems by distraction. PNAS. 2006 Aug;103(31):11778–11783. Available from: doi: 10.1073/pnas.0602659103 16868087 PMC1544246

[20] Packard MG, Goodman J. Factors that influence the relative use of multiple memory systems. Hippocampus. 2013;23(11):1044–1052. doi: 10.1002/hipo.22178 23929809

[21] Carpenter HS. A behavioral and electrophysiological investigation of different aptitudes for L2 grammar in learners equated for proficiency level [dissertation]. Washington D.C.: Georgetown University. http://hdl.handle.net/10822/558127

[22] Brill-Schuetz K, Morgan-Short K. The role of procedural memory in adult second language acquisition. Proceedings of the 36^th^ Annual Meeting of the Cognitive Science Society; 2014 Jul 23–26; Quebec City, Canada. p. 260–265. https://escholarship.org/uc/item/0dc7958r

[23] Morgan-Short K, Faretta-Stutenberg M, Brill-Schuetz KA, Carpenter H, Wong PCM. Declarative and procedural memory as individual differences in second language acquisition. Biling-Lang Cogn. 2014;17:56–72. doi: 10.1017/S1366728912000715

[24] Hamrick P. Declarative and procedural memory abilities as individual differences in incidental language learning. Learn Individ Differ. 2015;44:9–15. doi: 10.1016/j.lindif.2015.10.003

[25] Buffington J, Demos A, Morgan-Short K. The reliability and validity of procedural memory assessments used in second language acquisition research. Stud Second Lang Acq. 2021;43(3):635–662. doi: 10.1017/S0272263121000127

[26] Pili-Moss D. Cognitive predictors of child second language comprehension and syntactic learning. Lang Learn. 2021;71(3):907–945. doi: 10.1111/lang.12454

[27] Ettlinger M, Bradlow AR, Wong PCM. Variability in the learning of complex morphophonology. Appl Psycholinguist. 2014;35:807–831. doi: 10.1017/S0142716412000586

[28] Brooks PJ, Kwoka N, Kempe V. Distributional effects and individual differences in L2 morphology learning: determinants of L2 morphology learning. Lang Learn. 2017;67(1):171–207. doi: 10.1111/lang.12204

[29] Abrahamsson N, Hyltenstam K. Age of onset and nativelikeness in a second language: listener perception versus linguistic scrutiny. Lang Learn. 2009;59:249–306. doi: 10.1111/j.1467-9922.2009.00507.x

[30] DeKeyser RM. The robustness of critical period effects in second language acquisition. Studies Second Lang Acq. 2000;22:499–533. doi: 10.1017/S0272263100004022

[31] Dąbrowska E. Experience, aptitude and individual differences in language attainment: a comparison of L1 and L2 speakers. Lang Learn. 2019;69(S1):72–100. doi: 10.1111/lang.12323

[32] Morgan-Short K. A neurolinguistic investigation of late-learned second language knowledge: the effects of explicit and implicit conditions [dissertation]. Washington D.C.: Georgetown University; 2007.

[33] Pili-Moss D. Tracking the early stages of child and adult comprehension of L2 morphosyntax: a pilot study. JESLA. 2017;1(1):113–125. doi: 10.22599/jesla.25

[34] Rey A. L’examen psychologique dans les cas d’encéphalopathie traumatique. Arch Psychologie. 1941;28:215–285.

[35] Osterrieth PA. Le test de copie d’une figure complexe: contribution al’etude de la perception et de la memoire. Arch Psychologie. 1944;30:206–356.

[36] Tupler LA, Welsh KA, Asare-Aboagye Y, Dawson DV. Reliability of the Rey-Osterrieth complex figure in use with memory-impaired patients. JCEN. 1995;17(4):566–579. doi: 10.1080/01688639508405146 7593476

[37] Taylor LB. Scoring criteria for the Rey-Osterrieth complex figure test. In: Spreen O, Strauss E, editors. A compendium of neuropsychological tests. Administration, norms, and commentary. New York: Oxford University Press; 1998. p. 350–351.

[38] Mondini S, Mapelli D, Vestri A, Arcara G, Bisiacchi PS. Esame neuropsicologico breve 2. Milano, Italia: Raffaello Cortina Editore; 2011.

[39] Ryan JJ, Morris J, Peterson L. Test-retest reliability of the Wechsler Memory Scale, Form I. J Clin Psychol. 1981;37(4):847–848. doi: 10.1002/1097-4679(198110)37:4&lt;847::aid-jclp2270370429&gt;3.0.co;2-k 7309876

[40] Lum JAG, Gelgic C, Conti-Ramsden G. Procedural and declarative memory in children with and without specific language impairment. Int J Lang Comm Dis. 2010;45(1):96–107. doi: 10.3109/13682820902752285 19900077 PMC2826154

[41] Stark-Inbar A, Raza M, Taylor JA, Ivry RB. Individual differences in implicit motor learning: Task specificity in sensorimotor adaptation and sequence learning. J Neurophysiol. 2017;117(1):412–428. doi: 10.1152/jn.01141.2015 27832611 PMC5253399

[42] Farkas BC, Janacsek K, Nemeth D. The reliability of the Alternating Serial Reaction Time task. PsyArXiv. 2022. 10.3758/s13428-022-02038-5 PMC10794483 36604378

[43] Hedenius M. Procedural and declarative memory in children with developmental disorders of language and literacy [dissertation]. Uppsala, Sweden: University of Uppsala; 2013. http://uu.diva-portal.org/smash/get/diva2:638188/FULLTEXT01.pdf

[44] Howard DV, Howard JH, Japikse K, DiYanni C, Thompson A, Somberg R. Implicit sequence learning: effects of level of structure, adult age, and extended practice. Psychol Aging. 2004;19(1):79–92. doi: 10.1037/0882-7974.19.1.79 15065933 PMC1224741

[45] Bennett IJ, Madden DJ, Vaidya CJ, Howard JH Jr, Howard DV. White matter integrity correlates of implicit sequence learning in healthy aging. Neurobiol Aging. 2011 Dec;32(12):2317.e1–12. doi: 10.1016/j.neurobiolaging.2010.03.017 20452099 PMC2920348

[46] Wechsler D. Wechsler Adult Intelligence Scale–Fourth edition: Technical and interpretive manual. San Antonio, TX: Pearson Assessment; 2008.

[47] Baddeley A. The fractionation of working memory. P Natl Acad Sci. 1996;93(24):13468–13472. doi: 10.1073/pnas.93.24.13468 8942958 PMC33632

[48] Monaco M, Costa A, Caltagirone C, Carlesimo GA. Forward and backward span for verbal and visuo-spatial data: standardization and normative data from an Italian adult population. Neurol Sci. 2013;34(5):749–754. doi: 10.1007/s10072-012-1130-x 22689311

[49] Rosa EM, Leow RP. Awareness, different learning conditions, and second language development. Appl Psycholinguist. 2004;25(2):269–292. doi: 10.1017/S0142716404001134

[50] Bates D, Machler M, Bolker B. lme4: Linear mixed-effects models using s4 classes. 2011. http://cran.R-project.org/package=lme4.

[51] R Development Core Team. R: a language and environment for statistical computing. Vienna: R Foundation for Statistical Computing; 2018.

[52] Cohen J. Statistical power analysis for the behavioral sciences. Abingdon: Routledge;1988.

[53] Sánchez-Meca J, Marín-Martínez F, Chacón-Moscoso S. Effect-size indices for dichotomized outcomes in meta-analysis. Psychol Methods. 2003 Dec;8(4):448–67. doi: 10.1037/1082-989X.8.4.448 14664682

[54] Williams JN. Learning without awareness. Stud Second Lang Acq. 2005;27(2):269–304. doi: 10.1017/S0272263105050138

[55] Shanks DR, St. John MF. Characteristics of dissociable human learning system. Behav Brain Sci. 1994;17:367–447. doi: 10.1017/S0140525X00035032

[56] Rasch B, Born J. About sleep’s role in memory. Physiol Rev. 2013 Apr;93(2):681–766. doi: 10.1152/physrev.00032.2012 23589831 PMC3768102

[57] Segalowitz N. Cognitive bases of second language fluency. New York/London: Routledge; 2010.

[58] Suzuki Y. The role of procedural learning ability in automatization of L2 morphology under different learning schedules: an exploratory study. Stud Second Lang Acq. 2018;40(4):923–937. doi: 10.1017/S0272263117000249

[59] Green P, Mac Leod CJ. SIMR: an R package for power analysis of generalized linear mixed models by simulation. Methods Ecol Evol. 2016;7:493–498. doi: 10.1111/2041-210X.12504

[60] Morgan-Short K, Deng Z, Brill-Schuetz KA, Faretta-Stutenberg M, Wong PCM, Wong FCK. A view of the neural representation of second language syntax through artificial language learning under implicit context of exposure. Stud Second Lang Acq. 2015;37(s2):383–419. doi: 10.1017/S0272263115000030

