# Supplementary material for: Long-term memory predictors of adult language learning at the interface between syntactic form and meaning
Source: PLoS One. 2022 Oct 3;17(10):e0275061. doi: 10.1371/journal.pone.0275061 (PMC9529097; doi:10.1371/journal.pone.0275061)
Supplement: S1 File — (DOCX) [file pone.0275061.s001.docx]

**Supplementary materials 1.**

**Debriefing interview**

1) Pensi che alcune delle parole fossero speciali? Perché?

[Do you think any of the words of the new language were special? Why?]

2) Pensi che la nuova lingua che hai ascoltato avesse delle regole speciali? Per esempio?

[Do you think the new language you heard had any special rules? For example?]

3) Immagina che il/la tuo/a migliore amico/a voglia giocare a questo gioco. Adesso che sei un/a esperto/a, cosa potresti dirgli per aiutarlo/a a fare molti punti più velocemente?

[Suppose your best friend wanted to play this game. Now that you are an

expert, what would you tell him/her to help them make a lot of points quickly?]

4) C'è nient'altro che vorresti dirmi sulla nuova lingua o il gioco?

[Is there anything else you would like to tell me about the language or the

gaming experience?]
